# Effects of Pressure and Temperature on the Atomic Fluctuations of Dihydrofolate Reductase from a Psychropiezophile and a Mesophile

**DOI:** 10.3390/ijms20061452

**Published:** 2019-03-22

**Authors:** Qi Huang, Jocelyn M. Rodgers, Russell J. Hemley, Toshiko Ichiye

**Affiliations:** 1 Department of Chemistry, Georgetown University, Washington, DC 20057, USA; qh24@georgetown.edu (Q.H.); jrodgers78@gmail.com (J.M.R.); 2 Department of Civil and Environmental Engineering, George Washington University, Washington, DC 20052, USA; rhemley@email.gwu.edu

**Keywords:** protein dynamics, pressure effects, piezophile, hydrogen bonds

## Abstract

Determining the effects of extreme conditions on proteins from “extremophilic” and mesophilic microbes is important for understanding how life adapts to living at extremes as well as how extreme conditions can be used for sterilization and food preservation. Previous molecular dynamics simulations of dihydrofolate reductase (DHFR) from a psychropiezophile (cold- and pressure-loving), *Moritella profunda* (Mp), and a mesophile, *Escherichia coli* (Ec), at various pressures and temperatures indicate that atomic fluctuations, which are important for enzyme function, increase with both temperature and pressure. Here, the factors that cause increases in atomic fluctuations in the simulations are examined. The fluctuations increase with temperature not only because of greater thermal energy and thermal expansion of the protein but also because hydrogen bonds between protein atoms are weakened. However, the increase in fluctuations with pressure cannot be due to thermal energy, which remains constant, nor the compressive effects of pressure, but instead, the hydrogen bonds are also weakened. In addition, increased temperature causes larger increases in fluctuations of the loop regions of MpDHFR than EcDHFR, and increased pressure causes both increases and decreases in fluctuations of the loops, which differ between the two.

## 1. Introduction

Determining the effects of temperature and pressure on proteins is important for understanding how extremophiles adapt to thrive under extreme conditions as well as how to kill pathogenic microbes using temperature (i.e., pasteurization of foods) or pressure (i.e., “pascalization” or high-pressure processing of foods). Both temperature and pressure can cause the unfolding of proteins. Thermal unfolding has been studied for decades [1], and much is now understood about high hydrostatic pressure unfolding of proteins [2,3]. Pressure unfolding, which generally occurs above ~2 kbar, is apparently fundamentally different than thermal unfolding [4]. While thermal unfolding involves exposure of the inner hydrophobic core to the bulk solvent, pressure unfolding appears to involve extensive hydration in the interior of the protein [5]. In other words, pressure denaturation apparently occurs because the system will have a lower volume if water fills cavities inside the protein than if the cavities are compressed so that the more open solvated states become thermodynamically favored [6,7]. Supporting evidence includes high-pressure studies of crystal structures, which show that water is present in a large-cavity mutant of T4 lysozyme only at 1.5 kbar and above [8], and in cavities of mesophile isopropyl malate dehydrogenase (IPMDH) and dihydrofolate reductase (DHFR) only at 5 kbar and above [9,10]. Other differences include that the pressure unfolded state appears to be more compact than the thermally unfolded state [11] and that pressure unfolding is a slower process [12].

Besides folding and unfolding, the internal dynamics of folded proteins have been long recognized as important for protein function [13,14]. While the temperature effects on atomic fluctuations have been studied for decades [15], the effects of pressures up to ~1 kbar, which are pressures that organisms have been found at so far [16,17] but below that where unfolding of most proteins occurs [3], are less well-studied. At these pressures, simple compression of the protein [18] might be relevant. For instance, comparisons of crystal structures of homologous IPMDH and DHFR from a piezophile (pressure-loving) and a mesophile [19,20] indicate larger internal cavity volumes in the piezophile protein, suggesting that water penetration may be less of an evolutionary concern than compressibility. In addition, it has been pointed out that atomic fluctuations of a protein must be large enough to allow water to enter internal cavities for pressure unfolding to occur [21]. Interestingly, H/D exchange studies of apomyoglobin indicate that conformational fluctuations much smaller than local unfolding provide pathways for water to diffuse into the protein and that these fluctuations are enhanced by pressures up to 1.5 kbar [22].

Much has also been learned from studies of adaptations in homologous proteins from extremophiles and mesophiles that allow the microbes to thrive at their growth conditions, which have been called the “corresponding states”, where the proteins should behave similarly [23,24]. For instance, homologous enzymes tend to have similar stability and flexibility near their corresponding states since both are needed for activity [25,26]. However, increasing stability tends to increase rigidity while increasing flexibility tends to increase instability, so a balance is needed. Thus, stability at a particular extreme is not necessarily greater for an enzyme from an extremophile that lives under that extreme than from a mesophile if the need for flexibility is greater [25,26]. 

DHFR is one of the most thoroughly experimentally characterized enzymes from piezophiles, or more specifically, psychropiezophiles (cold and pressure loving) that are cold deep-sea microbes. For instance, the activity, stability, and structure of DHFR from the psychropiezophile, *Moritella profunda* (Mp), which has an optimal growth pressure (*P*_G_) of 220 bar at 6 °C [27], have been thoroughly compared with that of DHFR from the mesophile, *Escherichia coli* (Ec), which has an optimal growth temperature (*T*_G_) of 37 °C at 1 bar. MpDHFR has maximum enzyme activity at 500 bar while EcDHFR shows monotonic inactivation by pressure above 1 bar at 25 °C [20]. In addition, apo-MpDHFR has an unfolding pressure of 0.7 kbar [20] and temperature of 37.5 ± 0.8 °C [28] while apo-EcDHFR has an unfolding pressure of 2.7 kbar [20] and temperature of 51.6 ± 0.7 °C [29]. This marginal stability of enzymes from piezophiles has been noted for DHFR from other piezophiles as well as other enzymes from piezophiles [30]. This suggests that flexibility may be more important for these deep-sea enzymes, although this may be an adaptation for cold rather than for high pressure [24].

Our group has been examining the mean square fluctuations (MSF) of atomic coordinates in molecular dynamics (MD) simulations as a measure of the stability/flexibility balance needed for corresponding states behavior. Our simulations of MpDHFR and EcDHFR bound to tetrahydrofolate (THF) at various combinations of temperatures (25 or 37 °C) and pressures (1 or 220 bar) indicate that MSF, on a timescale of ~10 ns, averaged over the heavy atoms of the protein, are potential markers for corresponding states behavior because they become similar at the *P*_G_ and *T*_G_ of the organism from which the DHFR was isolated [31]. As expected, the MSF of the proteins in the simulations increases with temperature due to thermal energy. Interestingly, the MSF decrease slightly with pressure on a sub-nanosec timescale but actually increase with pressure on a nanosec-plus timescale [31]. 

To examine this further, our group has developed the quasiharmonic approximation for energy landscapes (QHAEL) method for analyzing pressure and temperature effects on proteins [32]. In this method, multiple short simulations generated on a grid of pressures and temperatures are used to explore the effective potential energy wells for a given protein atom created by its neighboring atoms. A quasiharmonic approximation is used to describe the average atomic fluctuations so that the average effects of temperature and pressure on a protein can be quantitated via an average force constant for the effective potential energy wells. The analysis can separate local versus collective motions by examining motion above and below the so-called protein glass transition, a dramatic change in protein dynamical properties that occurs at ~200 K [33]. Atomic motion is dominated by simple harmonic motion within an underlying local potential energy well below this temperature while the collective motion of groups of atoms becomes apparent above this temperature. QHAEL analyses of the above mentioned MD simulations of EcDHFR and MpDHFR [34] indicates that temperature widens the underlying local potential energy wells while pressure makes them steeper, which is consistent with the previous observation that the sub-nanosec timescale MSF decrease with pressure. In addition, the increased MSF at the nanosec-plus timescale at a higher pressure appears to be associated with collective motions that appear above the glass transition, although the physical origin was not apparent in this work. This suggests that sub-kilobar pressure may enhance flexibility by making collective modes more accessible, which is also consistent with experimental observations of pressure-induced unfolding [4] and pressure induced conformational shifts [35]. Also, the analysis showed that the underlying local potential energy wells (i.e., below the glass transition) of MpDHFR are softer than those of EcDHFR.

The increased atomic fluctuations at 220 bar were somewhat surprising since 2 μs molecular dynamics simulations of another protein, [4Fe-4S] ferredoxin, at 1 bar, 2 kbar, 5 kbar, 8 kbar, and 10 kbar showed decreasing atomic fluctuations with increasing pressure [36]. While the latter simulations are not inconsistent with high-pressure unfolding, which is a slow equilibrium process, the small fluctuations even on the *μ*sec timescale beg the question as to why the folded state does not become kinetically trapped in a metastable state if the fluctuations only decrease with increasing pressure. Thus, the physical origin of the increased fluctuations at 220 bar is of interest, especially since on the single protein length-scale, pressure fluctuations are in the kbar range [37]. A clue may come from the experimentally-observed increase in the diffusion coefficient of water with pressure followed by a decrease, with a maximum somewhat under ~2 kbar [38,39,40,41]. Interestingly, the maximum is pronounced near 5 °C but becomes smaller with increasing temperature and disappears by ~40 °C. The increase in the diffusion coefficient has been attributed to pressure distorting hydrogen bonds between water molecules [39].

Finally, a question for simulations of biomolecules at states away from where they were parameterized is the applicability of the parameters used in the force fields. Many simulations of proteins with different biomolecular force fields have been performed at high pressures, mainly between 5 and 20 kbar, to understand pressure unfolding [42]. Simulations of bovine pancreatic trypsin inhibitor with two different force fields at 5 kbar gave opposite results for pressure effects on backbone hydrogen bond lengths, so only one was in agreement with NMR experiments [43], although the changes were so small that it is difficult to assess the significance. In addition, these studies were performed before significant improvements to the backbone potential [44] were implemented in most biomolecular force fields. Moreover, the pressures in the abovementioned simulations of EcDHFR and MpDHFR [31] are an order of magnitude smaller than the ones used to examine pressure unfolding, so they probe the potential at states much closer to where it was parameterized. In these simulations, the CHARMM36 all-atom nonpolarizable potential energy parameter set with an updated backbone potential [44,45] was used for the proteins. In addition, by TIP4P-Ew [46], which gives good pressure- and temperature-dependent properties for liquid water [47], was used to model water.

Here, the effects of pressure and temperature on the MSF are studied in molecular dynamics (MD) simulations of MpDHFR:THF and EcDHFR:THF. Since the effects of pressure and temperature are coupled, i.e., the behavior of the diffusion coefficient of water mentioned above, the proteins are considered at various combinations of the *P*_G_ and *T*_G_ of the organisms Mp and Ec. Since the specific adaptations for pressure and temperature are addressed elsewhere [48], the main interest here is in the physical changes in the protein leading to the increased MSF at high pressure at the same temperature observed previously in simulations [31] since the thermal energy is constant. While the role of water in the pressure unfolding of a protein has been established, the role in increasing fluctuations of the folded protein is not clear. Since pressure acts on both the protein and the surrounding water, water can only be “pushed” inside the protein if the forces holding the folded protein together can be overcome, such as when the atomic fluctuations are large enough. This suggests that factors other than water inside the protein may be responsible for the large MSF at sub-kbar pressures in the previous simulations. A variety of factors are examined, including the volume per heavy atom, the number of water molecules inside the protein, and the number and lifetime of hydrogen bonds. The hydrogen bond lifetimes can assess the strength of the hydrogen bonds. The results indicate that both pressure and temperature generally weaken intra-protein hydrogen bonds. In addition, the effects of pressure and temperature on the two proteins are different from each other and between proteins, despite the structural similarity of the proteins. Increased temperature causes increased fluctuations in the loop regions, with larger increases in MpDHFR than EcDHFR. On the other hand, increased pressure causes both increases and decreases fluctuations in the loops, with opposite trends in EcDHFR and MpDHFR.

## 2. Results and Discussion

The effects of pressure and temperature on the properties of EcDHFR and MpDHFR in molecular dynamics (MD) simulations are presented here. Various average properties were examined (Table 1). The average mean-square fluctuations of the protein heavy atoms (MSF) and the average apparent volume per heavy atom (*V*_app_*/N*_HA_) from our previous work [31] are given to demonstrate the trends that were observed, while the average number of water molecules inside the protein (*N*_w,in_), the average number of hydrogen bonds (*N*_HB_), and the hydrogen bond lifetimes (*τ*_HB_) from the calculations here are presented as possible physical causes of the trends seen in the MSF and *V*_app_*/N*_HA_. These are first discussed briefly in terms of differences between EcDHFR and MpDHFR. In these simulations, both proteins were below their experimental unfolding pressures at 220 bar, although MpDHFR:THF at 310 K is close to the experimental unfolding temperature of MpDHFR:DHF, which is 316.15 ± 0.3 K [28]. At a given pressure and temperature state, MpDHFR has greater motion (larger MSF), more room per heavy atom to move around in (larger *V*_app_*/N*_HA_), more water inside the protein (larger *N*_w,in_), and fewer hydrogen bonds (smaller *N*_HB_) compared to EcDHFR. In addition, although the *τ*_HB_ appear generally very slightly shorter in MpDHFR than EcDHFR, the differences are not statistically significant, and so the number *N*_HB_ is more important. The fewer hydrogen bonds in MpDHFR means that the protein is held together less tightly, so that *V*_app_*/N*_HA_ is larger, which consequently gives more room for the atoms to move so the MSF are larger. The larger MSF and greater *V*_app_*/N*_HA_ also allows more water inside the protein, so *N*_w,in_ is larger. These factors would make MpDHFR more subject to high pressure and high temperature unfolding, which is consistent with the experimental results of the lower unfolding pressure [20] and temperature [28,29] of MpDHFR relative to those of EcDHFR described in the Introduction. Thus, it appears that MpDHFR is adapted for flexibility at cold temperature rather than stability at high pressure. 

Next, the changes in the properties of the proteins are examined when the temperature increases from 279 K (*T*_G_ of Mp) to 310 K (*T*_G_ of Ec) at constant pressure (Table 2). As temperature increases, the average MSF increase by ~45% for EcDHFR and ~80% for MpDHFR at either pressure. The proteins expand very slightly since *V*_app_*/N*_HA_ increases by ~3% because thermal fluctuations give rise to thermal expansion. Also, *N*_w,in_ increases by less than one water molecule for both except for MpDHFR at 220 bar. The latter is likely not significant but rather due to the definition of water inside the protein, which is less well-defined for MpDHFR at 310 K because it becomes less structured as it approaches its melting temperature. More importantly, the increases in *N*_w,in_ do not appear to be enough to affect the entire protein and appears to be a consequence of the increased atomic fluctuations and greater room per heavy atom. Finally, the decreases in *N*_HB_ and *τ*_HB_ indicate that the hydrogen bonds are weakened with increasing temperature, which is expected because of the greater thermal energy for breaking hydrogen bonds.

Thus, the causes of increased MSF with temperature in the simulations can be ascribed as follows. First, increased thermal energy will increase atomic fluctuations even if the local potential energy wells do not change. Second, thermal expansion will make the underlying potential energy wells shallower and therefore increase atomic fluctuations because the atoms are further apart. Third, relatively weaker hydrogen bonds at the higher temperatures will make the potential energy wells softer because the interactions with the neighboring atoms are reduced. Interestingly, breaking hydrogen bonds is a transitional behavior that requires sufficient energy; i.e., such as might occur above the glass transition temperature. In addition, because the hydrogen bonds appear weaker with respect to temperature in MpDHFR than in EcDHFR, the increase in the MSF is larger in MpDHFR.

Now, the changes in the properties of the proteins are examined when the pressure increases from 1 bar (*P*_G_ of Ec) to 220 bar (*P*_G_ of Mp) at constant temperature (Table 3). As pressure increases for both EcDHFR and MpDHFR, the average MSF also increase (by ~10%) but the proteins remain constant in size since *V*_app_*/N*_HA_ remain approximately constant, indicating essentially no compressive effects since the change in pressure is relatively small. There is also very little change in *N*_w,in_ except for a slight decrease of about one water molecule in MpDHFR at 310 K, which again is likely not significant but rather due to the definition of *N*_w,in_. Again, the changes in *N*_w,in_ do not appear to be enough to affect the entire protein. Finally, the hydrogen bonds appear to weaken with increasing pressure since *τ*_HB_ becomes shorter. However, since *N*_HB_ stays the same or becomes larger in MpDHFR, the weakening of the hydrogen bonds may not be uniformly distributed.

Thus, the causes of increased MSF with pressure in the simulations can be ascribed as follows. First, thermal energy cannot be a cause of increased MSF since these pressure changes are at constant temperatures. Second, pressure tends to compress the underlying potential energy wells [34] so that the direct effect of pressure makes the wells steeper, which would cause the MSF to decrease. However, weaker hydrogen bonds will make the local potential energy wells softer and the protein more flexible. Because of the suggested importance of weakening of hydrogen bonds by pressure on the MSF, the hydrogen bond lifetimes will be examined further below.

**Figure 1:**
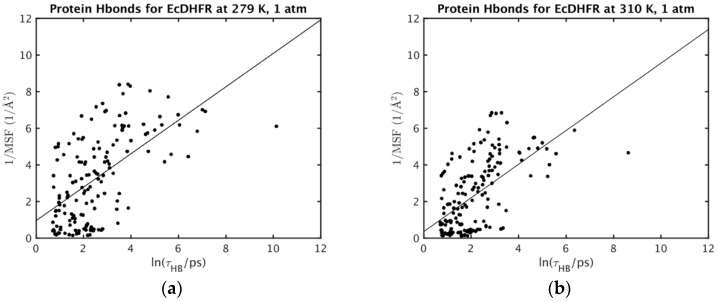
Correlation of inverse atomic fluctuations of hydrogen bond acceptors with the associated hydrogen bond lifetimes longer than 2 ps for EcDHFR at 1 bar and (**a**) 279 K or (**b**) 310 K.

**Figure 2:**
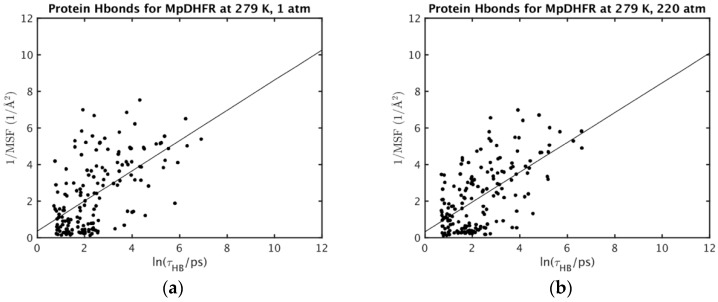
Correlation of inverse atomic fluctuations of hydrogen bond acceptors with the associated hydrogen bond lifetimes longer than 2 ps for MpDHFR at 279 K and (**a**) 1 or (**b**) 220 bar.

The correlations of the MSF of a hydrogen bond acceptor with the average *τ* for the acceptor hydrogen bonded to any donor (Equation (2)) are now examined. While perfect correlation cannot be expected, scatter plots of 1/MSF versus ln *τ* (Figure 1 and Figure 2; results for other conditions are given in Figure S1 for MpDHFR and Figure S2 for EcDHFR of the Supplementary Materials) show that the atomic fluctuations tend to be inversely correlated with the lifetimes, which is consistent with shorter hydrogen lifetimes leading to larger fluctuations. In addition, the plots of 1/MSF versus ln *τ* for EcDHFR at 279 and 310 K (Figure 1a,b) illustrate that increasing the temperature leads to overall both shorter lifetimes and larger fluctuations while maintaining the trend for an inverse correlation between the two. However, the plots of 1/MSF versus ln *τ* for MpDHFR at 1 and 220 bar (Figure 2a,b) illustrate that increasing the pressure to 220 bar maintains the inverse correlation between lifetimes and fluctuations, but the net increase in MSF can be accomplished by making some smaller and some larger.

**Figure 3:**
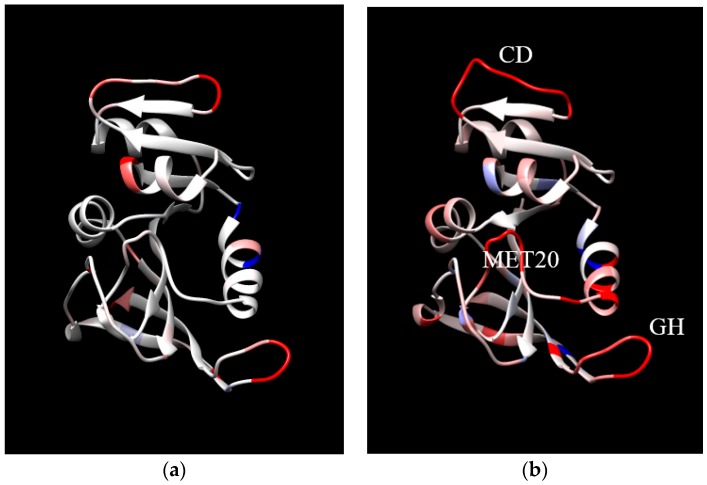
The difference in mean square fluctuations (MSF) between 279 and 310 K at 1 bar for (**a**) EcDHFR and (**b**) MpDHFR. The coloring of each residue indicates the difference in the average MSF of all heavy atoms in the residue, where the coloring scale is that –0.5 is dark blue, 0 is white, and 2.0 is dark red. Loops are identified in (**b**).

**Figure 4:**
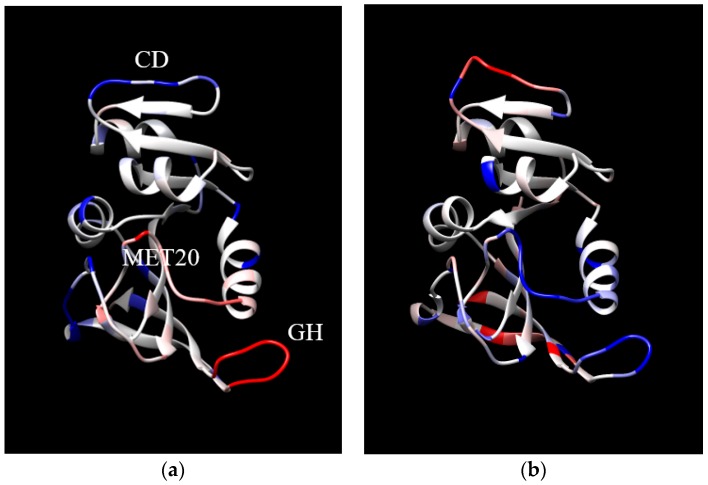
The difference in MSF between 1 and 220 bar at 279 K for (**a**) EcDHFR and (**b**) MpDHFR. The coloring of each residue indicates the difference in the average MSF of all heavy atoms in the residue, where the coloring scale is that –0.5 is dark blue, 0 is colored, and 2.0 is colored. Loops are identified in (**a**).

Finally, the location in the protein of the changes in MSF with increases in temperature and pressure are examined. For increases in temperature at 1 bar, the MSF increase in similar locations for EcDHFR and MpDHFR, but the changes are larger in MpDHFR (Figure 3a,b). The MSF become larger mainly in the loops, specifically the CD loop at the top and the GH loop at the bottom right. In addition, the Met20 loop in the center of Figure 3b also has a large increase. On the other hand, for increases in pressure at 220 bar, the changes in MSF are much different for EcDHFR and MpDHFR (Figure 4a,b). For instance, the CD loop becomes more rigid in EcDHFR while it becomes more flexible in MpDHFR. In addition, the Met20 and GH loops become more flexible in EcDHFR while they become more rigid in MpDHFR. Thus, increased temperature tends to increase the MSF of loops at the surface because thermal energy is distributed evenly throughout the protein and surface loops have more freedom to move. On the other hand, increased pressure can either increase or decrease the MSF of the loops because the available volume for atomic fluctuations is dependent on the local sequence, which can differ even in homologous proteins. Since the opening of a loop means water is penetrating between the loop and the rest of the protein so that the loop becomes solvated, small increases in pressure may cause a loop to close, while larger increases in pressure may favor water penetration between the loop and the rest of the protein. Moreover, the magnitudes of the pressures involved will depend on the sequence-dependent packing between the loop and the rest of the protein.

## 3. Materials and Methods 

The simulations analyzed here have been previously reported [31], so the simulation methods are described briefly here and additional details can be found in the previous work. The starting coordinates for the proteins in the previous work were obtained from the Protein Data Bank (PDB) [49] for MpDHFR (PDB ID: 2ZZA) [50] and EcDHFR (PDB ID: 1RX2) [51] with THF built in. MD simulations and other coordinate manipulations were performed using the molecular mechanics package CHARMM version 37b2 and version 40b2 [52]. The set-up was performed in CHARMMing [53] using default protocols except as noted. The CHARMM36 all-atom nonpolarizable potential energy parameter set [44,45] was used for the proteins. In addition, water was modeled by TIP4P-Ew [46], which gives good properties for liquid water in the range of temperatures and pressures examined here [47]. Additionally, a force field for THF was generated using the CHARMM Generalized Force Field server [54]. The simulations utilized the leapfrog Verlet algorithm with a time step of 0.001 ps and were maintained in the *NPT* ensemble with the Nosé-Hoover algorithm [55] for the thermostat and barostat. Periodic boundary conditions and the particle mesh Ewald (PME) summation algorithm with a *k*-space grid spacing of ~0.34 Å [56,57] were used. The rhombic dodecahedral simulation box had a distance between faces of ~71 Å, and each protein was in 0.15 M KCl, so that there are 7414 water molecules, 26 K^+^, and 15 Cl^−^ for MpDHFR, and 7420 water molecules 27 K^+^ and 14 Cl^−^ for EcDHFR. After heating for 100 ps and pressurizing the 220 bar simulations for 22 ps, the simulations were allowed to equilibrate unperturbed for 4 ns after which the 50 ns production run was collected. Coordinates were written every 1 ps, and averages were calculated at this interval except as noted.

Averages of various properties were calculated from the 50 ns simulations of DHFR in aqueous solution. The time averages, denoted by angle brackets, were over the entire 50 ns, and the errors were from block averages of 10-ns intervals except as noted. The average MSF of protein heavy atoms were calculated as the average over all protein heavy atoms *i* of 〈Δ*r_i_*^2^〉, which is the block average of the fluctuations with respect to the average position over 10-ns intervals over the five 10-ns intervals.

(1) $$\mathrm{MSF}=\frac{1}{N_{\mathrm{HA}}}\overset{N_{\mathrm{HA}}}{\underset{i=1}{\sum }}\langle \Delta r_{i}^{2}\rangle$$

where *N*_HA_ is the number of heavy atoms of the protein. The apparent volume of protein *V*_app_ was given by

(2) $$V_{\mathrm{app}}=\langle V_{\mathrm{MD}}\rangle -N_{w}\langle V_{w}\rangle \;$$

where 〈*V*_MD_〉 is the average volume of the simulation box, *N*_w_ is the total number of water molecules in the simulation, and 〈*V*_w_〉 is the average molecular volume of a water molecule from a simulation of the pure solvent at the same pressure and temperature. The *V*_app_ per heavy atom, *V*_app_/*N*_HA,_ is thus a measure of the average volume available to each heavy atom. Water molecules were defined to be inside the protein if they were within 4.00 Å of any protein heavy atom and with no other water molecules within 3.36 Å. The average number of water molecules inside the protein, *N*_w,in_, was calculated using coordinates at intervals of 100 ps in the entire 50 ns period. Other possible measures of volume, such as the effective volume inside a defined surface for a protein structure, were not used because the apparent volume can be measured experimentally and also measures the effects of fluctuations in the structure.

Hydrogen bonds were defined as having a distance between the donor hydrogen *i* and acceptor atom *j* smaller than 2.40 Å and the angle of D-H^…^A larger than 130°. Here, the donor or the acceptor could be a charged moiety. The average number of hydrogen bonds, *N*_HB_, is the block average of the number of hydrogen bonds at each timestep. The average lifetime, *τ_ij_*, of a hydrogen bond between *i* and *j* is given by the average of

(3) $$\tau_{ij}=\frac{1}{n_{ij}}\overset{n_{ij}}{\underset{i=1}{\sum }}t_{ij}\left(n\right)$$

in which *t_ij_* was the total time that a hydrogen bond is continuously formed between *i* and *j* and *n_ij_* was the number of events in the 50 ns. This simple measure of lifetime was used instead of using the decay of the hydrogen bond auto-correlation function [58] often used in liquids [59] because many types of hydrogen bonds are found in a protein, which may range in lifetime from ps to more than 10 ns, making the auto-correlation function method impractical. In our other studies of aqueous solutions [60], Equation (3) gives lifetimes that are somewhat shorter than those from auto-correlations but otherwise exhibit the same trends. Since our interest is in relative changes, not absolute values, this definition was chosen. Ultimately, the best definition depends on the experimental or theoretical quantity of interest, and our interest is as an indicator of hydrogen bond strength. The average lifetime for hydrogen bond pairs was defined as:

(4) $$\tau_{\mathrm{HB}}=\frac{1}{N}\underset{i,j}{\sum }\tau_{ij}$$

where *N* is the total number of unique hydrogen bonds found at any time during the simulation, which differs from *N*_HB_. Hydrogen bond pairs with lifetimes greater than 1 ns at 279 K were left out of the average over all protein-protein hydrogen bond pairs (but included in the average for *N*_HB_) because statistics are insufficient. The excluded pairs are Thr113 Oγ1 to Asp27 Oδ1, Leu8 N to Thr113 O, and Tyr111 N to Leu4 O in EcDHFR, which are apparently adaptations for higher temperatures [34]. Two hydrogen bonds simultaneously formed with the same protein atom were calculated as two separate events. 

## 4. Conclusions

The physical origins of the increases in atomic fluctuations observed in the MD simulations of MpDHFR and EcDHFR upon increases in temperature and pressure have been investigated here. Of the possible causes of the larger fluctuations as the temperature is increased at constant pressure, increased thermal energy, thermal expansion, and weakened intra-protein hydrogen bonds all appear to contribute to increased fluctuations. However, the same cannot be said for why pressure increases fluctuations at a constant temperature since the thermal energy is constant and the underlying local atomic potential wells are compressed [34]. Instead, the simulations indicate that pressure weakens intra-protein hydrogen bonds, as evidenced by shorter lifetimes. Since the increased *N*_w,in_ is less than 1 between 220 and 310 K and less than 0.15 between 1 and 220 bar, it seems unlikely that the entry of so little water could cause the observed increases in the MSF, so the slightly increased *N*_w,in_ appears to be a consequence of the larger atomic fluctuations. However, this has yet to be examined in simulations of other proteins or experiments at these kinds of pressure conditions. In addition, it is reminiscent of the changes in the diffusion coefficient of water under pressure [38,39,40,41] described in the Introduction.

In summary, atomic fluctuations of folded proteins can provide insight into the mechanical properties of proteins at different temperatures and pressures, which can be useful in understanding how life adapts to extreme conditions, as well as how extreme conditions can be used for sterilization and food preservation. Of course, the conclusions here are for two homologous proteins, one from a mesophile and one from a piezophile, so other studies are needed to examine whether these findings are general. For instance, it is necessary to examine other proteins, as well as proteins from other microbes. Also, microbes have been found to grow at temperatures up to 122 °C and at pressures up to 1.1 kbar, so greater ranges of both temperature and pressure need to be examined. However, the differences in the responses of the mesophile and piezophile DHFR to pressure indicate that large effects may be more localized to the specific sequence. In particular, since pressure both can increase fluctuations by weakening hydrogen bonds and decrease them by compressing the underlying potential energy wells, the balance between the two opposing effects is likely to differ between proteins, even homologous proteins. This means that the net effect of an increase in fluctuations with pressure observed here for DHFR is not necessarily universal for all proteins, although the weakening of hydrogen bonds would tend to increase the fluctuations.

## Figures and Tables

**Table 1:** Average properties for EcDHFR and MpDHFR in the molecular dynamics (MD) simulations.

Protein (*T*, K; *P*, bar)	MSF (Å^2^) *	*V*_app_*/N*_HA_ (Å^3^) *	*N*_w,in_	*N*_HB_	*τ*_HB_ (ps)
EcDHFR (279; 1)	0.65 ± 0.02	17.38 ± 0.02	1.96 ± 0.31	107 ± 2	27.7 ± 3.4
MpDHFR (279; 1)	0.76 ± 0.08	17.73 ± 0.04	3.25 ± 0.30	103 ± 2	30.6 ± 3.7
EcDHFR (279; 220)	0.71 ± 0.11	17.55 ± 0.01	2.08 ± 0.31	105 ± 1	25.5 ± 2.7
MpDHFR (279; 220)	0.83 ± 0.04	17.77 ± 0.03	3.28 ± 0.40	104 ± 1	22.5 ± 1.8
EcDHFR (310; 1)	0.93 ± 0.07	17.87 ± 0.02	2.36 ± 0.37	106 ± 1	11.6 ± 1.2
MpDHFR (310; 1)	1.39 ± 0.15	18.26 ± 0.02	4.21 ± 0.32	101 ± 2	11.2 ± 0.6
EcDHFR (310; 220)	1.04 ± 0.14	17.82 ± 0.01	2.47 ± 0.36	104 ± 1	11.2 ± 1.1
MpDHFR (310; 220)	1.49 ± 0.16	18.25 ± 0.01	3.26 ± 0.42	101 ± 2	11.1 ± 1.1

* From reference [31].

**Table 2:** Changes of average properties for EcDHFR and MpDHFR with temperature at constant pressure *P*, where Δ*_T_* indicates a change from 279 to 310 K and percentages are with respect to the 279 K value.

Protein (*P*, bar)	Δ*_T_*%MSF *	Δ*_T_*%*V*_app_*/N*_HA_ *	Δ*_T_N*_w,in_	Δ*_T_N*_HB_	Δ*_T_*%*τ*_HB_
EcDHFR (1)	43	3	0.40	−1	−58
EcDHFR (220)	46	2	0.39	−1	−56
MpDHFR (1)	83	3	0.96	−2	−63
MpDHFR (220)	80	3	−0.02	−3	−51

* Based on data from reference [31].

**Table 3:** Changes of average properties for EcDHFR and MpDHFR with pressure at constant temperature *T*, where Δ*_P_* indicates a change from 1 to 220 bar and percentages are with respect to the 1 bar value. Note that MpDHFR is close to its melting temperature at 310 K.

Protein (*T*, K)	Δ*_P_*%MSF *	Δ*_P_*%*V*_app_*/N*_HA_*	Δ*_P_N*_w,in_	Δ*_P_N*_HB_	Δ*_P_*%*τ*_HB_
EcDHFR (279)	9	1	0.12	−2	−8
EcDHFR (310)	12	0	0.11	−2	−3
MpDHFR (279)	9	0	0.03	1	−26
MpDHFR (310)	7	0	−0.95	0	−1

* Based on data from reference [31].

## Supplementary Materials

Supplementary materials can be found at https://www.mdpi.com/1422-0067/20/6/1452/s1.
