# Supplementary material for: Effects of Pressure and Temperature on the Atomic Fluctuations of Dihydrofolate Reductase from a Psychropiezophile and a Mesophile
Source: Int J Mol Sci. 2019 Mar 22;20(6):1452. doi: 10.3390/ijms20061452 (PMC6470811; doi:10.3390/ijms20061452)
Supplement: Supplementary file 1 [file ijms-20-01452-s001.pdf]

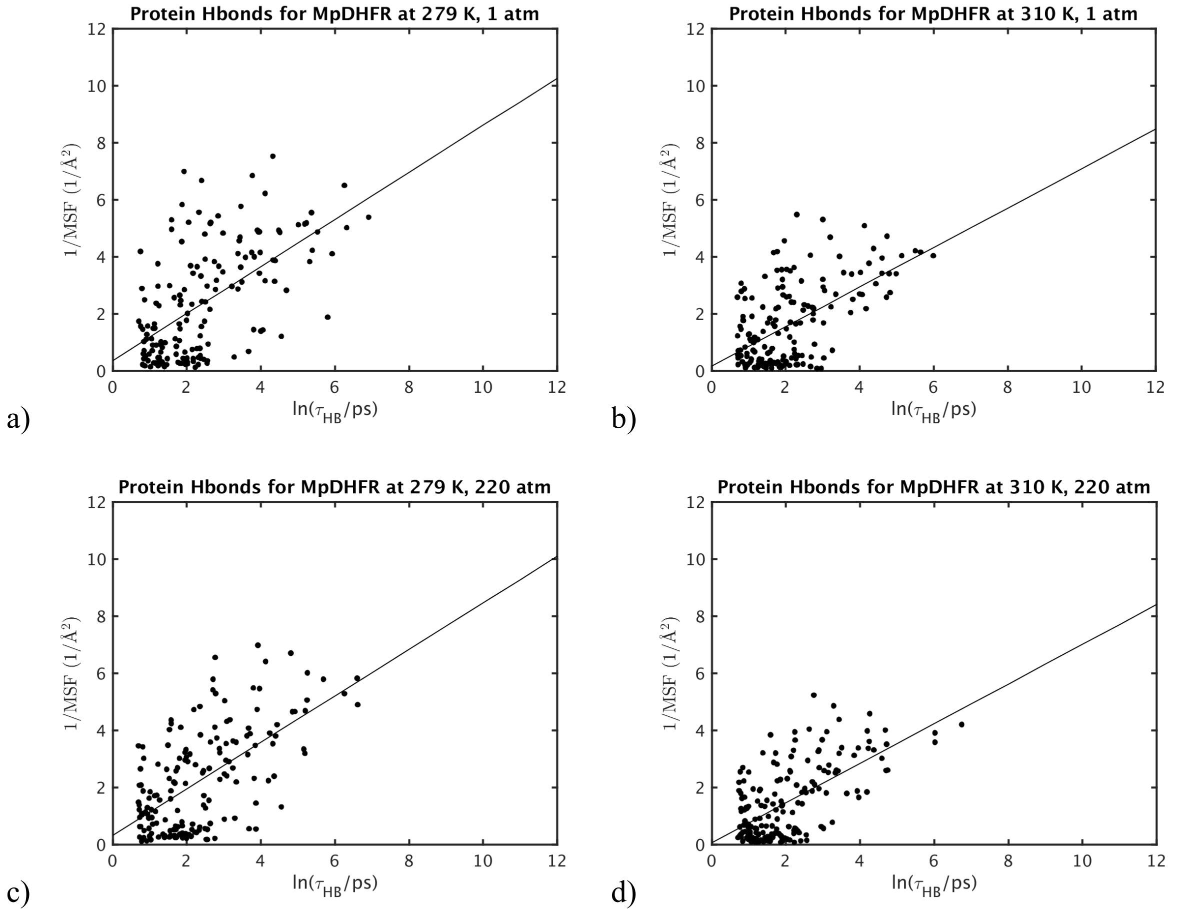

Figure S1. Correlation of inverse atomic fluctuations of hydrogen bond acceptors with the associated hydrogen bond lifetimes longer than 2 ps for MpDHFR at different conditions. Correlation coefficients: a) 0.584, b) 0.575, c) 0.611, d) 0.623.

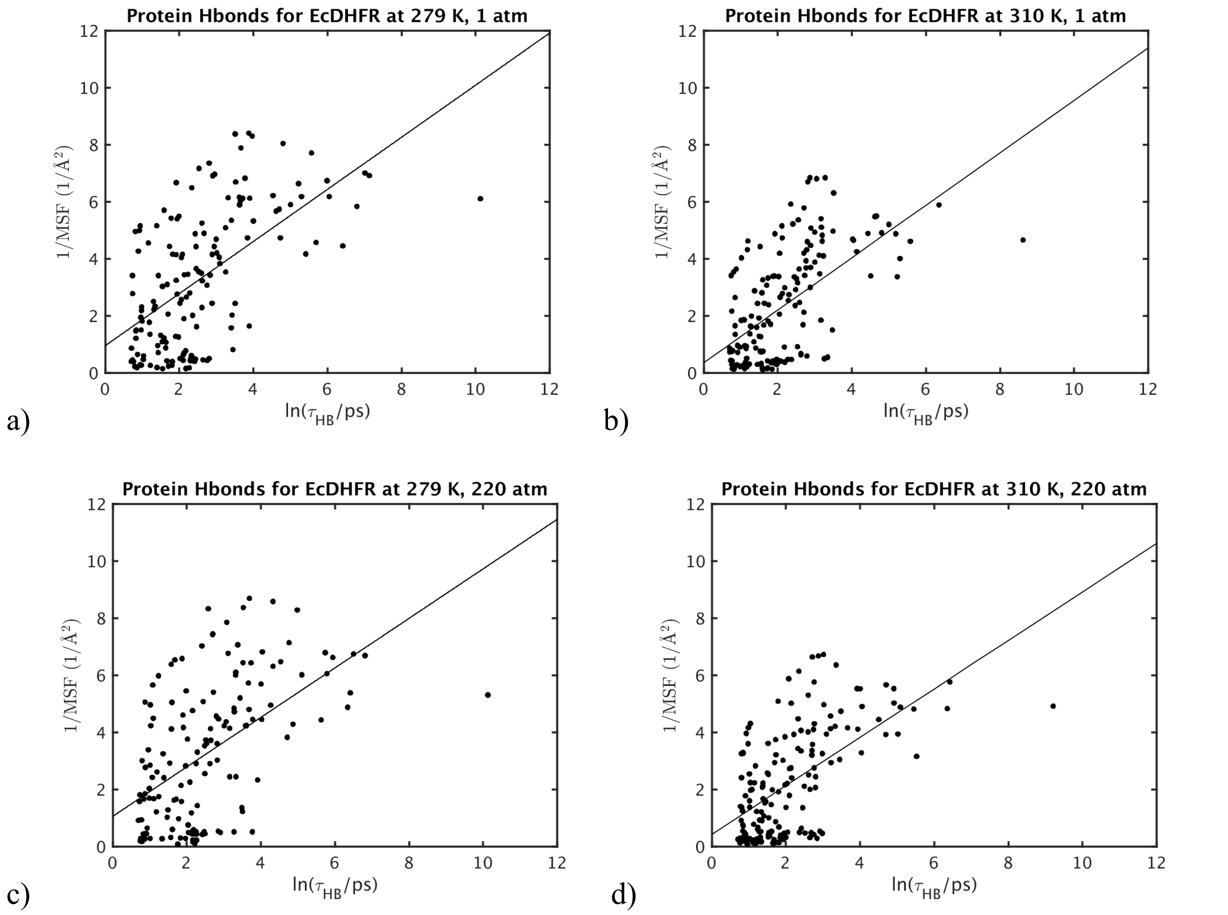

Figure S2. Correlation of inverse atomic fluctuations of hydrogen bond acceptors with the associated hydrogen bond lifetimes longer than 2 ps for EcDHFR at different conditions. Correlation coefficients: a) 0.594, b) 0.588, c) 0.535, d) 0.592.
